# Aesthetic Intervention: Lines of Flight

**DOI:** 10.1177/1940844720968200

**Published:** 2021-05

**Authors:** Susan Mackay, Gabriel Soler, Tessa Wyatt

**Affiliations:** 1 3124 Department of Counselling Studies and Applied Social Sciences, University of Edinburgh, UK; 2 65932 Independent Scholar and Artist, Edinburgh, UK

**Keywords:** art as activism, inquiry as activism, line of flight, new materialism

## Abstract

During the European Congress of Qualitative Inquiry, Edinburgh, 2019, we offered an esthetic intervention: two spaces open to delegates in which they could explore and express their interactions with the conference through the assemblage of paper, paint, crayons, scissors, glue, glitter, bodies, breath, memories, thoughts—ineffable and effable. Delegates were invited to produce either individual journals, individual pieces, or contribute to large collective pieces of art. In this article, we follow the lines of flight to create the event and reflect on the process that led up to and continued after the esthetic intervention.

## Introduction

*It starts with a line. A line on the page. A line of thought. A line of flight.*

*A line which pulls together and dissents.*

*Then multiplies and disperses*.

In this article, we, Susan, Gabriel, and Tess, follow a line that traces the project *Create Art*, which we developed for the European Conference of Qualitative Inquiry (ECQI), Edinburgh, 2019.

Initially, we follow the line of inception, when the idea arose. We explore the process to construct an environment for the aesthetic intervention. This moves us to reflect on the one-day drop-in art space facilitated during the conference where we contemplate the activating space, intensities, and art media. Last, we review a moment of shared activism after the conference, when the three authors responded to the artworks that had been produced. Throughout this paper, we reflect with an implicit view of new materialism (Barad, 2003; Bennet, 2010; Braidotti, 2006), the notion of inquiry as activism, along with the Deleuzian concept of line of flight.

Together, we write making different lines become a composition. Our lines are not the same, they are woven to create a story, they cannot capture or offer a concrete sequence or results. The lines of creation, thought, and action were thrown upwards, hover, and shift elsewhere. Invisible motion, and emotion. This hesitation, movement, and dispersal are reflected in the structure of this paper. We offer gestures, images, and moments from the process.

In the sections that follow, we narrate the story of our intervention Create Art. In “Incipient Lines,” we describe the moment we first thought of the idea of having a space for art in a conference. Then in the following section, we narrate how we structured and planned the event. The “Creative Lines” section is the story of being in the conference when delegates came to the art room we had prepared. In the section on “Activating Lines,” we reflect on our artistic intervention and the idea of activism. In the following section, we show what happened after the event, when we three artists and organizers gathered to reflect on the experience. The concluding section has our final thoughts summarizing the whole assemblage of events.

## Incipient Lines

Leaving the British Autoethnography Conference 2018:

A line, a train track. Two lines, parallel tracks. An early start, sleep in our eyes as the train pulls us faster toward London. The incessant and erratic motion of the train matches our conversation. We are imagining, conjuring a place, separate from but part of an imagined future qualitative conference. A place for expression. A place for Art. A different form of expressing from sitting and listening, or conversation post presentations. A space where art materials and material bodies digest, process and express the unsaid, unsayable content, and haecceity of conferences. The ineffable alongside rational engagement. A place for drawing lines of connections, atmosphere, place. Milieu.

## Hidden Lines

The idea, the intangible imaginary, we pulled along the tracks toward London, emerged as a concrete offer of a room at the ECQI, Edinburgh, 2019, to facilitate art expression for the 300 or so delegates.

Coming from different backgrounds, we, Tess, Gabriel, and Susan, share a passion for art practices. Our original aim to offer art at a conference comes from this simple fact: we want to share. Art for us is a place of research, of inquiry. We engage with it as a not-yet-known place. Acknowledge the pulse of each medium and feel into the dialogue with materials. There is a thought-in-formation that takes shape. It is used as part of our scholarship, and as an element of the process of meaning making. We are excited to be able to offer this to academics in the *wordy* environment of a conference.

**Figure 1:**
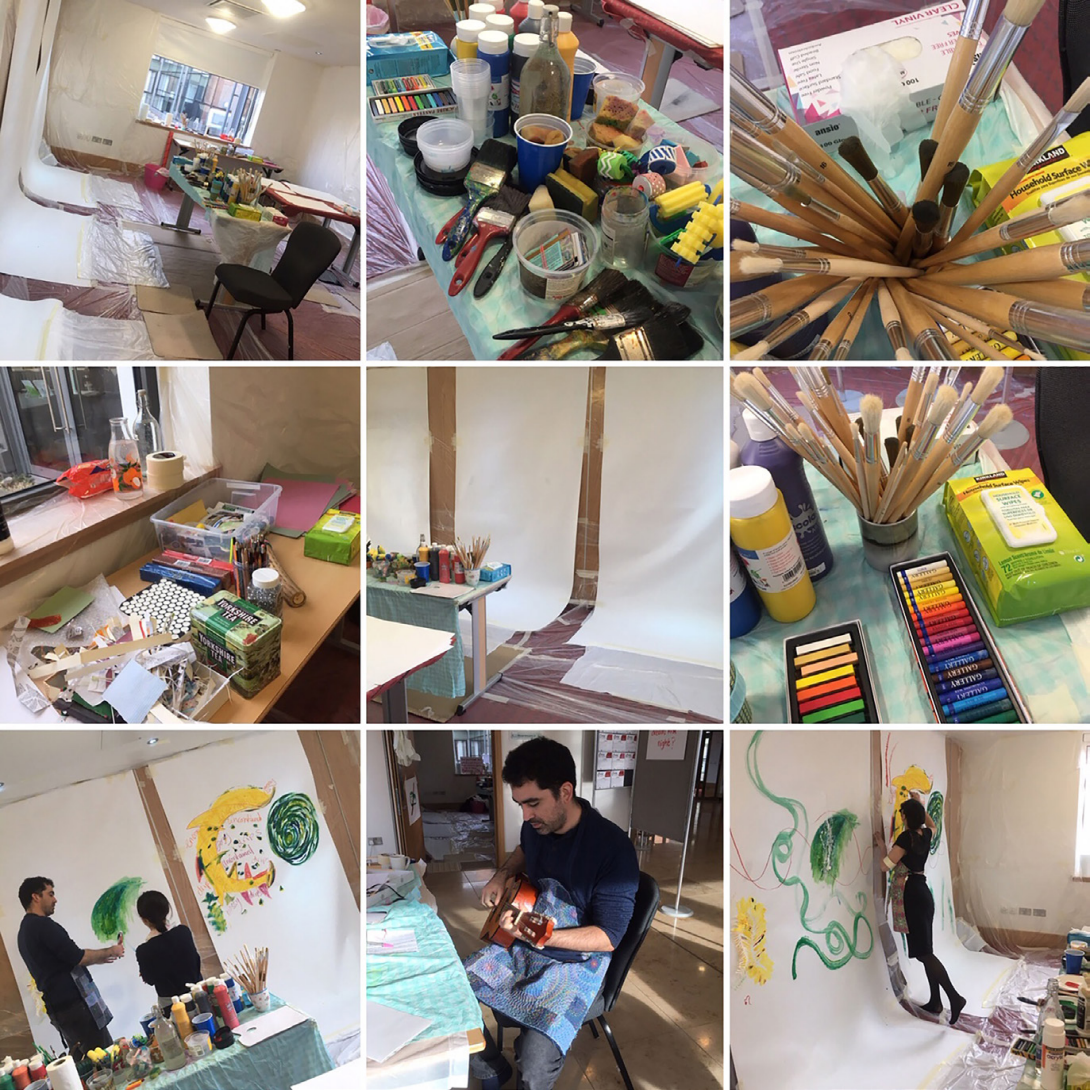
Create art. (Photo credit: Co-author Tessa Wyatt)

But art making is not all about gestures of sliding paint. To create an effective space for delegates to respond with art materials requires planning and thought. Attention to minutiae. It requires structure and thinking, to produce a careful boundary to facilitate focused unhindered creativity.

We order supplies. Plan how to manage imagined hordes of keen creatives. Discuss how to divide the spaces. Produce pamphlets and flyers.

It’s the day before the conference starts and we are tight with anticipation. A wound-up energy releases through the demands of the physical activity, moving boxes from cars, upstairs, along corridors. Standing, legs akimbo, we contemplate our task to make a conference room suitable for creativity—seeping art materials and mobile bodies. We pull reams of giddy plastic to cover the pristine white walls and compact carpet. The noise of tape pulling and sticking resonates with the loud whispers of the carefree plastic sheeting. Along with the inaudible sound of our concentration. We move tables, situate, anticipate, create. Plastic rips, we compensate. Laugh, worry, drink tea, eat biscuits and chocolate.

The line has split; we are not artists, we are planners. Constructing. Using our knowledge and experience to pull a hidden line underneath what will be seen and experienced by the participants. An unseen line, a frame, that will support their experience. A structure to hold the potential of spontaneity and creativity.

## Creative Lines

Our art invitation to the delegates has three strands: large collective pieces, smaller individual pieces, and art journaling. We have access to two spaces to offer this: the foyer for journaling, and a conference room for painting. We facilitate these spaces throughout 1 day of the conference, inviting delegates to respond to research presentations, conversations, words, feelings, thoughts—the Assemblage of ECQI.

In the conference room, there are large sheets of paper taped against the wall, from floor to ceiling, sheets of sugar or construction paper, collage materials—fragments, glitter, scissors, and glue. Large bottles of paint, sponges, brushes, rollers, and pastels. The space is designed for relaxing expression, creation, discovery. The foyer has tables with a curious supply of materials and prefabricated journal books.

People arrive and we are ready. Tess guards the entrance with aprons, instructions, and little journals on lanyards to be used for reflection and to manage numbers. Susan and Gabriel offered options: *are you up to something big and collective or something smaller and personal?*

There is a sense of playful attention. Focus generating creativity. Smoothed and undulating concentration. Gabriel strums his guitar, singing. Bodies move, paint smears and drips on paper.

**Figure 2:**
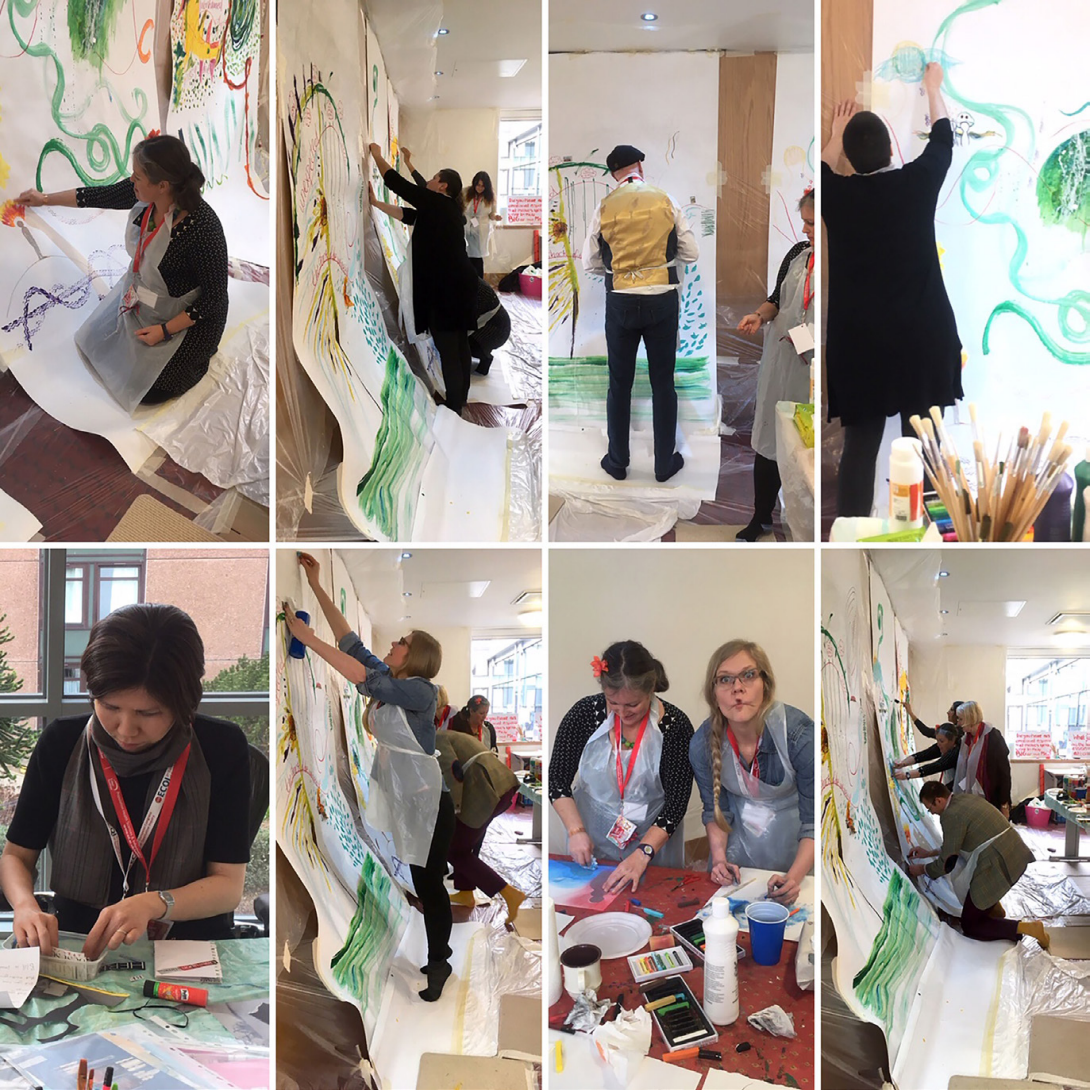
Working in create art. (Photo Credit: Co-author Tessa Wyatt)

*Inspired by the woman beside him using her hands to push paint over the large piece of paper, D takes his socks and shoes off and paints with his foot… and then asks for a picture because his wife will not believe him. N wants to make a* Pollock *splash, an exuberance not matching her usually composed manners. B is slowly explaining the connection between her two drawings with masking tape “because it represents how things in my life were separate, but there is a line that connects from the past to the future.”*

## Activating Lines: Space for Material Intensities

When we work on the relationships between our practice and activism, we feel that the word *activism* is too *major* for what we did. Our invitation was *minor* (Manning, 2016) in the sense of not following an art manual but creating a space that could allow improvisation to happen. We aimed to create a space for affect and intensities to come, for interactions to emerge. The space, a frame for something *new*, something we could not anticipate. More than activism, we think of *activation*. Our aim was to generate a place where people are *activated*. Understanding *activation* as an activism without predecided direction. By activating during the conference, we offered participants a different way to produce qualitative data. This is the sensual data of Richardson and St. Pierre (2005).

Art media affect us, as we affect them. Materials are *lively matter* that invite us to action. They are not passive, but motion-full and responsive; they have agency. For example, when the paper slid away from the wall, it undulated, creating an unexpected cavity, between paper, plastic, and wall. A unique material interaction. They move and we move them, and we are moved. One line invites the next one. Or like the paint belies paper, and crayons aggravate paint. One color calls or resists another color. The blank space existed momentarily … and then lines appeared entangling, entwining, resisting, isolating, connecting. The unification of location, materials, bodies, breath, actions, thoughts, dreams, paint, glue, glitter, tools, affects and intensities, all started from a calling by the paper to be marked … and it was.

**Figure 3:**
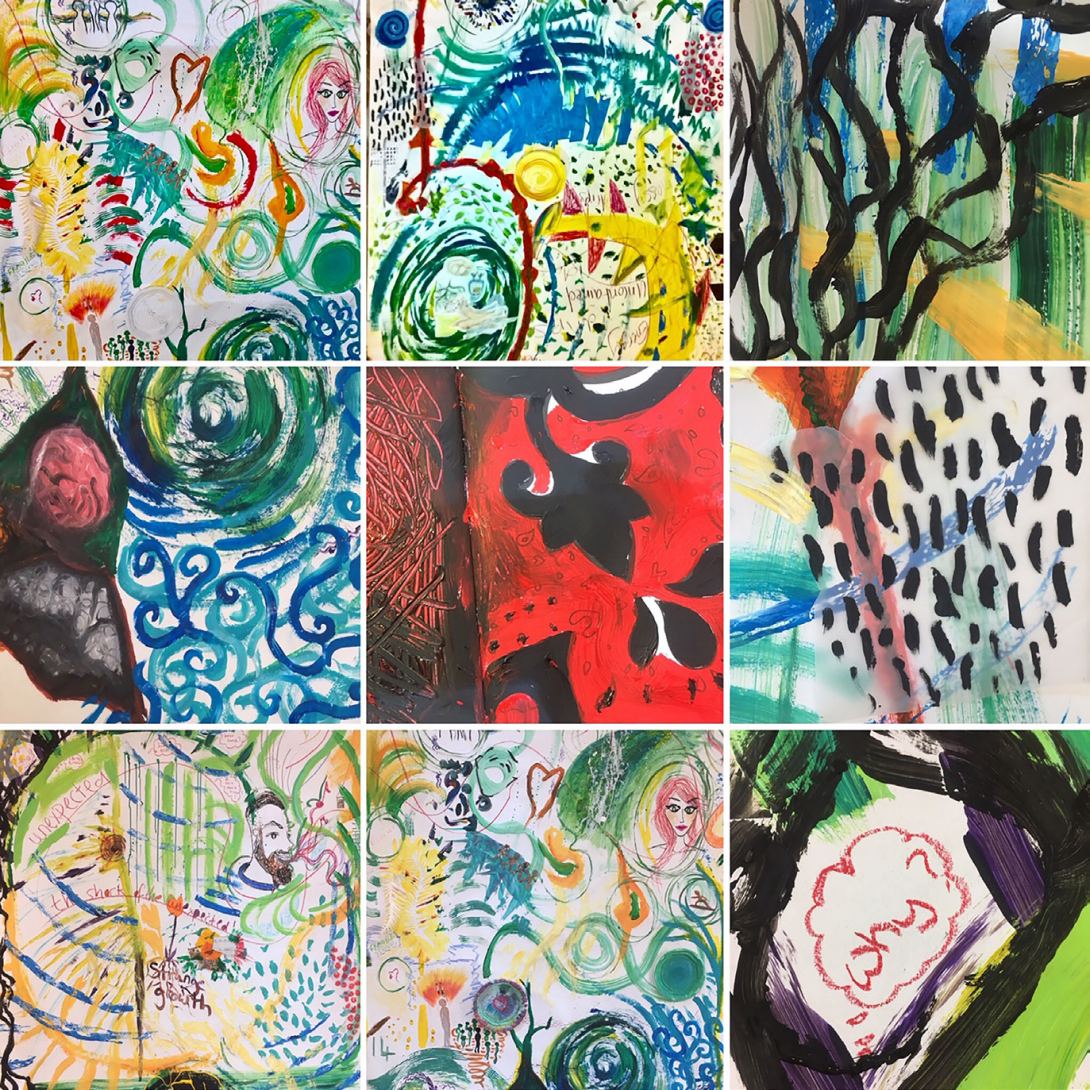
Sections of the create art panels. (Photo credit: Co-author Tessa Wyatt)

Sometimes a direction emerges, something new that pushes the gestures to go forward, to go away of the known territories. A line of flight.

Lines of flight are connecting all the machines of the room (what we usually call tools, object, or living beings). Lines of flight make the paint assemble with the brush, assemble with the hand, assemble with the paper, assemble with the paint. The multiplicities that constitute us rise and meet the multiplicities that constitute other.

So many intensities collected, so many stories the conference-territory opened. Each of us as a multiplicity creating multiplicities. As Deleuze and Guattari (1988) explain, “multiplicities are defined by the outside: by the abstract line, the line of flight or deterritorialization according to which they change in nature and connect with other multiplicities” (p. 9). Multiplicity is the central ontology of Deleuze, the word that describes everything that exists as a constant becoming, as something always being different to itself: there is no static essence or stable identity. Lines of flight are described as processes of deterritorialization (going out of the original assemblages), which makes a *multiplicity* enter in an *abstract* engagement that allows “changes in nature” and new connections to other multiplicities.

In the art room, “each of these becomings brings about the deterritorialization of one term and the reterritorialization of the other” (Deleuze & Guattari, 1988, p. 10); therefore, one intervention on the page goes out of their original configuration (deterritorialization) and engages with the work of the other (deterritorialization). Hence, the room was full of different lines of flight reaching each other. One gesture would deterritorialize to reterritorialize the image that was made by another gesture. As this process follows, “the two becomings interlink and form relays in a circulation of intensities pushing the deterritorialization ever further” (1988, p. 10), that is, pushing our collective work even further.

In this way, we understand the process of *activation* as a space where lines of flight become together (changing in nature and connecting with each other). Our art as *activation* aims to produce no-predetermined configurations in the social field through art practices.

The space incites new relations and actions: activity. Activation. The frame holds intensities—moments reflected in the gestures on the papers. Intensities are sometimes hard to catch. Intensities come as an affect we feel in the body and/or with the body. Intensities are tensions, calls, motions. They come and go. We create a context for them to stay a bit longer, for them to come and inhabit us, and move us to create a line… a line of flight, a line where the intensities are made into a visual event—a mark on the page. We create a frame, and inside the frame exploration, improvisation emerges. Disrupting the usual chat between sessions and, instead, offered a different dialogue. Making the response to a qualitative conference also a qualitative expression.

It is late on a Thursday. We roll the papers, we store the pots, brushes, paint. We get rid of the plastic that covered the room for the playing-with-materials. We observe what we have, happy to have seen an idea become an event, the engagement, the pleasure in the faces of people making art as part of a conference.

## Dissenting Lines: Letting Rip

It’s a month after the conference. We meet to consider, to review, and to decide. The art production from the day is abundant. Now contemplating it, there are large billowing and creaking rolls of paper, journals, and flat pieces of paper, lying between us and twinkling spilled glitter in Susan’s front room. What to do with all this art? Tess folds the paper and rips. There is a sensation of release as the tears follow their own itinerant journey. As if the intensities from the conference are shimmering outwards. Gabriel takes scissors, and cuts. We destroy, disrupt, and create something else. We look at the awkward shapes we have created through our destruction and see anew. From a different frame, we see new motions, new connections, and relationships. We have moved the line onwards, as if it had continued hesitating in the air, and now we are creating a secondary esthetic intervention. Breaking the whole, but following movement, following action. Destroying to create.

**Figure 4:**
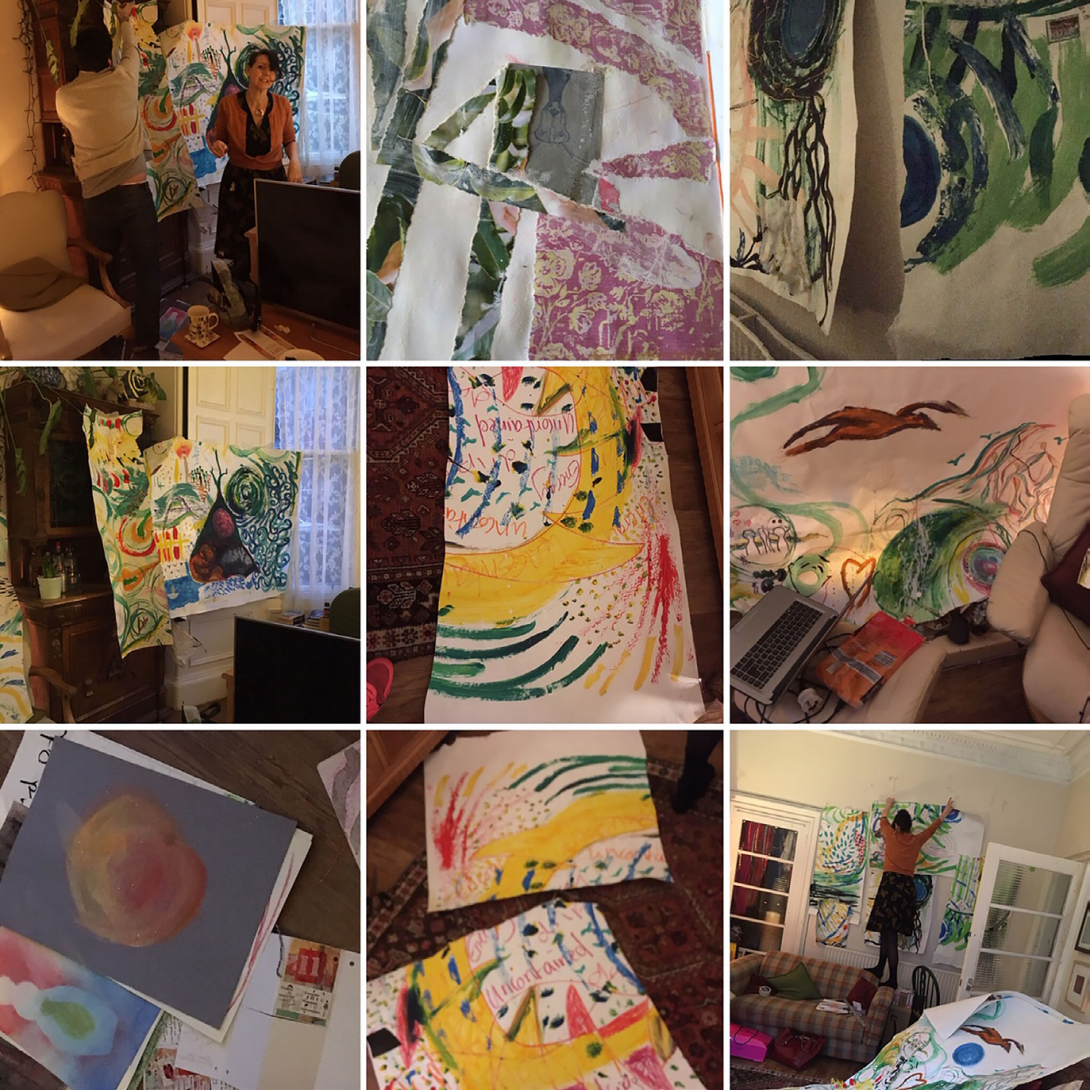
Destroying to create. (Photo Credit:Co-authorTessa Wyatt)

We create new responses to the responses, to the responses. Not through representation but following the line of material affect. We are affected by this art. We rip up, then construct words, following multiple lines which move out of the frame.

The line disperses and multiplies.

We move to our computers, interchanging emails. Feel the distance from the physical engagement. Separate from our own engagement with paper and paint. We work with memories; we intellectualise what already happened. The activation that emerged on that day keeps resonating with us. Inviting us to create new spaces for the intensities to come. New playgrounds where the intensities take our bodies to create art.

## Concluding Lines

A line that started in a train travels underground to become a frame for a space with art materials inside the ECQI. Converging lines that extend to a multitude of lines of flight: ongoing rippling affects by those who created, those who witnessed the activity. The large collectively painted on sheets of paper, the smaller individual pieces, and the intimacy of the art journals.

The line then dissents, as we three who had created the event allowed the painted paper its mastery, as we let rip, producing jagged lines and straight lines, which cut through the apparent passivity of the image. We destroyed to activate the complexity of art, creation, order, destruction.

We consider art as a qualitative space that wrecks against any categorization. It is a full gestalt that cannot be translated into another medium. But insists on its own forces. We do not try to represent what we did with the words, but to share part of the process we had as the organizers. We think the images work by themselves: they create new intensities, new lines that cannot be translated into words.

The space we created disrupted the written word, which is perceived as the foremost way of presenting thought and research. Our space was productive, creating a different engagement with what we understand as a conference. We sense that the creative space worked together with the presentations, making new assemblages and opening other dialogs. As a nonguided place, it becomes an opportunity for explorations, new associations, wonderings/wanderings.

The line we follow is art activism through destruction, fragmentation, creation and construction. Therefore, we challenge the idea of the unitary and open a place to multiplicities. A line that starts as one breaks into many lines of flight becoming together.

*The lines*

*Still sing at night*

*The songs we painted*

**Figure 5:**
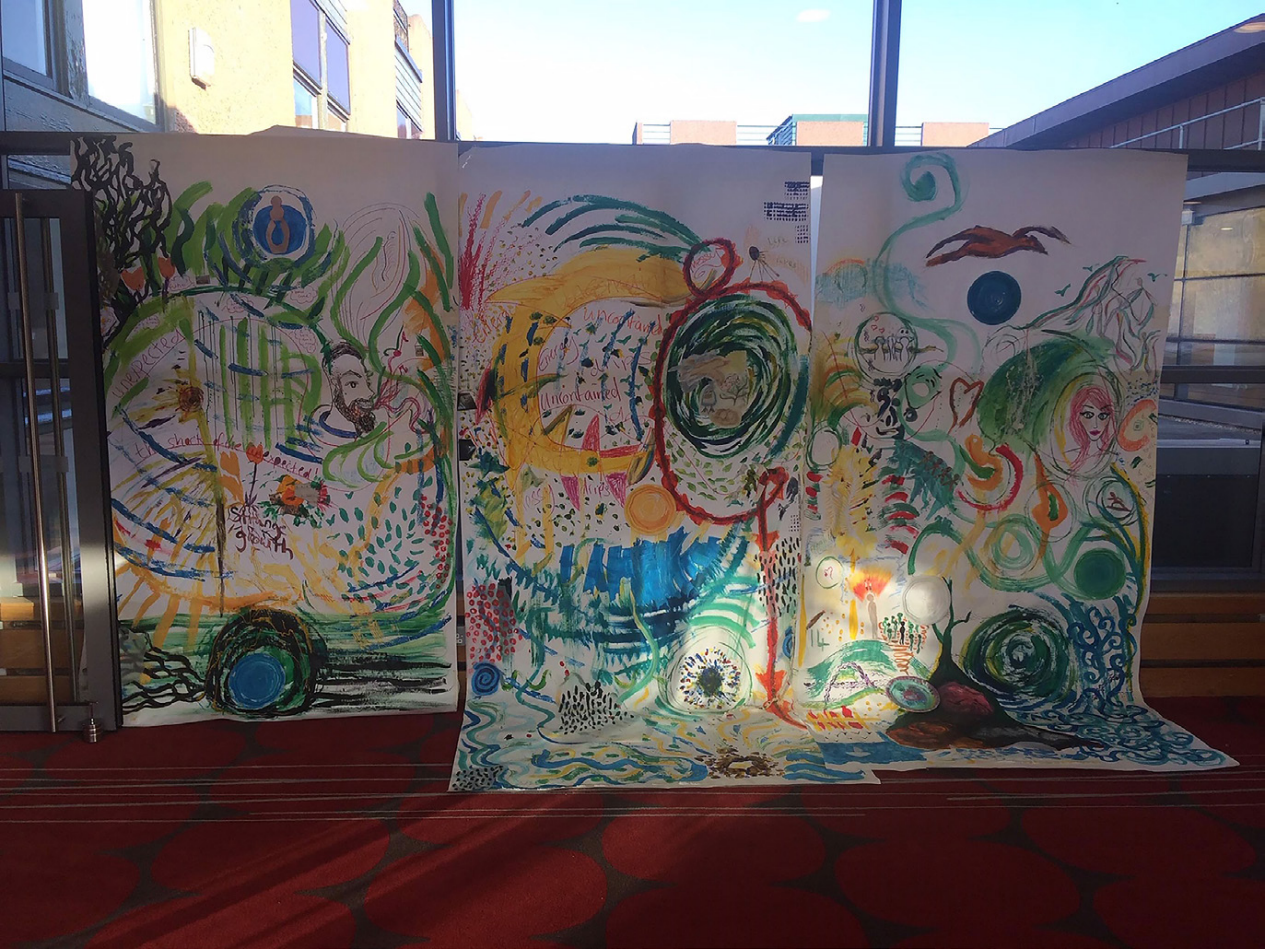
Three panels on display. (Photo Credit: Co-author Susan Mackay)
